# Downregulation of miR-320a/383-sponge-like long non-coding RNA NLC1-C (narcolepsy candidate-region 1 genes) is associated with male infertility and promotes testicular embryonal carcinoma cell proliferation

**DOI:** 10.1038/cddis.2015.267

**Published:** 2015-11-05

**Authors:** M Lü, H Tian, Y-x Cao, X He, L Chen, X Song, P Ping, H Huang, F Sun

**Affiliations:** 1 International Peace Maternity & Child Health Hospital, School of Medicine, Shanghai Jiaotong University, Shanghai, China; 2 Reproduction Medical Center, Department of Obstetrics and Gynecology, the First Affiliated Hospital of Anhui Medical University, Hefei, Anhui, China; 3 Shanghai Key Laboratory for Reproductive Medicine, Shanghai, China; 4 Institute of Biochemistry and Cell Biology, Shanghai Institutes for Biological Sciences, Chinese Academy of Sciences, Shanghai, China; 5 School of Life Sciences, University of Science and Technology of China, Hefei, Anhui, China; 6 Renji Hospital, School of Medicine, Shanghai Jiaotong University, Shanghai, China

## Abstract

Long non-coding RNAs (lncRNAs), which are extensively transcribed from the genome, have been proposed to be key regulators of diverse biological processes. However, little is known about the role of lncRNAs in regulating spermatogenesis in human males. Here, using microarray technology, we show altered expression of lncRNAs in the testes of infertile men with maturation arrest (MA) or hypospermatogenesis (Hypo), with 757 and 2370 differentially down-regulated and 475 and 163 up-regulated lncRNAs in MA and Hypo, respectively. These findings were confirmed by quantitative real-time PCR (qRT-PCR) assays on select lncRNAs, including HOTTIP, imsrna320, imsrna292 and NLC1-C (narcolepsy candidate-region 1 genes). Interestingly, NLC1-C, also known as long intergenic non-protein-coding RNA162 (LINC00162), was down-regulated in the cytoplasm and accumulated in the nucleus of spermatogonia and primary spermatocytes in the testes of infertile men with mixed patterns of MA compared with normal control. The accumulation of NLC1-C in the nucleus repressed miR-320a and miR-383 transcript and promoted testicular embryonal carcinoma cell proliferation by binding to Nucleolin. Here, we define a novel mechanism by which lncRNAs modulate miRNA expression at the transcriptional level by binding to RNA-binding proteins to regulate human spermatogenesis.

Infertility is a global reproductive health issue affecting approximately one in six couples attempting pregnancy worldwide. Half of the cases are due to male factors, and 75% of the patients are diagnosed as idiopathic since the molecular mechanisms underlying the defects remain unknown.^1^ A significant proportion of male infertility is accompanied by the clinical characteristics of men with uniform testicular maturation arrest (MA) and nonobstructive azoospermia (NOA) or severe oligozoospermia.^2^ Therefore, elucidating the underlying pathogenesis of MA may help improve treatment outcomes in these patients.

Spermatogenesis is a complex developmental programme that supports the generation of spermatozoa and fertility throughout the adult male life. Spermatogenesis can be divided into three principal phases: mitotic proliferation of spermatogonia, meiosis of spermatocytes, and haploid differentiation of spermatids,^3^ which are all strictly regulated by a complex transcriptional network. In addition to protein-coding messenger RNAs, many non-coding RNAs, including Dicer-dependent microRNAs (miRNAs),^4, 5, 6, 7, 8, 9^ endogenous small interfering RNAs (siRNA),^10^ PIWI-interacting RNAs^11, 12^ and long non-coding RNAs (lncRNAs)^13, 14^ also play a keyl role in regulation of genes during the process of spermatogenesis. However, the regulatory mechanisms of altered miRNAs and lncRNAs levels and functions still remain elusive.

miRNA biogenesis proceeds from primary miRNA transcripts that are transcribed from the host genome by RNA polymerase II. Primary miRNAs are further processed into mature miRNAs, which are eventually loaded into the RNA-induced silencing complex (RISC), leading to translational repression and mRNA degradation of their targets.^15^ Recent high-throughput sequencing technology has shown that thousands of long non-coding transcripts are actively transcribed from the human genome, as well as from other organisms.^16, 17^ lncRNAs are operationally defined as RNA transcripts that are longer than 200 nt but do not appear to have protein-coding potential.^18, 19, 20, 21, 22^ This class of RNAs includes intergenic non-coding RNAs (ncRNAs), pseudogene transcripts and many antisense RNAs.^18, 23^ Compared with their protein-coding counterparts, lncRNA genes are composed of fewer exons, under weaker selective constraints during evolution, and in relatively lower abundance. In addition, the expression of lncRNAs is strikingly cell type and tissue specific and, in many cases, even primate specific. Similar to protein-coding genes, lncRNAs have been implicated in diverse biological processes, including cell proliferation,^24, 25^ differentiation,^20, 22, 26, 27^ migration,^24, 28, 29^ immune response^30, 31^ and apoptosis,^32^ all of which have been implicated in tumorigenesis. In addition to being highly deregulated in tumours,^33^ lncRNAs have been found to act as tumour suppressors or oncogenes.^23, 28, 34, 35^ However, the role of lncRNAs in the process of human spermatogenesis has not yet been elucidated.

Here, we investigated a long intergenic non-coding RNA, NLC1-C (narcolepsy candidate-region 1 genes), which was significantly down-regulated in testicular tissues of NOA patients with MA. NLC1-C, the expression of which is restricted to spermatogonia and early spermatocytes, was significantly down-regulated in MA patients. These findings suggest that NLC1-C may act in the early stages of spermatogenesis, and thus may regulate germ cell proliferation or death. In this study, based on microarray technology and experimental approaches, we suggest that NLC1-C functions as a repressor to regulate the expression of miR-320a and miR-383 by binding to Nucleolin in the nucleus and in the cytoplasm, NLC1-C is the direct target of miR-320a and miR-383 in human spermatogenesis. However, when NLC1-C was accumulated in the nucleus of spermatogonia and primary spermatocytes in the testes, decreasing miR-320a and miR-383 expression, it leads to male infertility.

## Results

### The expression and the location of NLC1-C changes in MA patients and is associated with infertility

**Figure 1:**
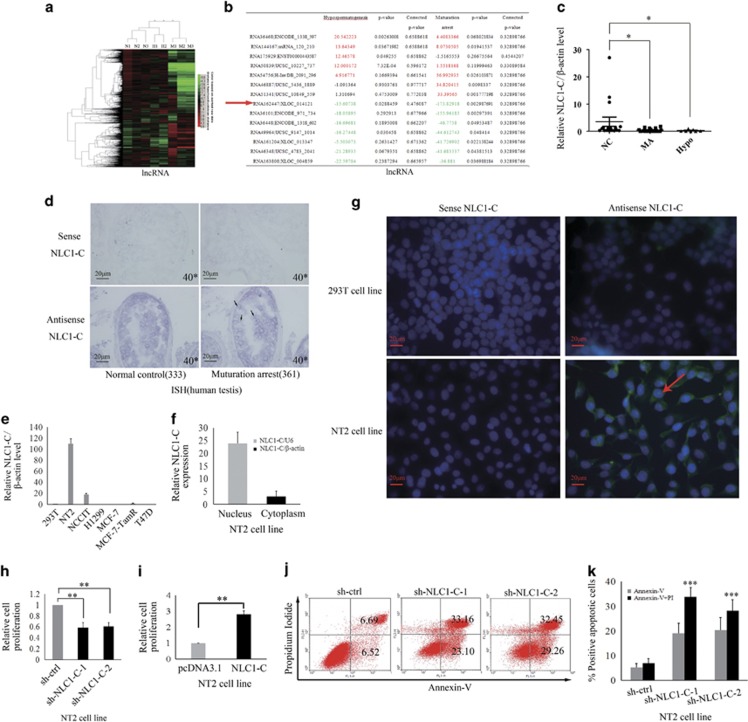
NLC1-C is down-regulated in NOA (MA and Hypo) patients and silencing of endogenous NLC1-C represses NT2 cell proliferation and promotes cell apoptosis. (**a**) The cluster heat map shows lncRNAs with an expression change fold >4 from microarray data (*P*<0.025) in NOA patients compared with controls. N, controls; H, Hypo patients; M, MA patients. (**b**) The data provided in the table represent the lncRNAs that were found to be desregulated significantly in NOA patients compared with controls. NLC1-C in the table is marked with a red arrow. (**c**) Quantitative real-time PCR analysis confirmed the microarray data: NLC1-C was down-regulated in NOA patients. (**d**) Localisation of NLC1-C in the testes of normal controls and maturation arrest patients by DIG-labelled RNAs by *in situ* hybridisation. The accumulation of NLC1-C in the nucleus is indicated by the black arrow. (**e**) Quantitative real-time PCR detection of NLC1-C expression in indicated human cell lines. Data are normalised to *β*-actin expression and HEK293 T (293 T) was set to a value of 1. (**f**) NLC1-C was localised in both the nucleus and cytoplasm of NT2 cells. Quantitative real-time PCR examination of NLC1-C expression in both the nucleus and cytoplasm of NT2 cells. Data are normalised to U6 expression in the nucleus and *β*-actin expression in the cytoplasm, respectively. (**g**) RNA FISH assay of NLC1-C localisation in 293T cell and the testicular embryonal carcinoma cell line NTERA-2 (NT2). Scale bars, 20 *μ*m. DAPI, 4′,6-diamidino-2-phenylindole. (**h** and **i**) Silencing and overexpression of NLC1-C inhibited and promoted cell proliferation. (**j** and **k**) Silencing of endogenous NLC1-C promoted cell apoptosis (**j**) and the proportions of cells apoptotic rates was shown in (**k)**. Proportions of cells apoptotic rates were determined using flow cytometry. Error bars indicate S.E.M. (*n*=3). *P<0.05, **P<0.01, ***P<0.001 (two-tailed Student's *t*-test), compared with negative controls

By employing a highly sensitive, high-throughput, and specific core lncRNA chip V2.0 microarray platform, the lncRNA expression profiles for testicular tissues were determined from three patients with MA, two patients with hypospermatogenesis (Hypo) and three controls. A total of 757 and 2370 differentially down-regulated and 475 and 163 up-regulated lncRNAs were found in MA and Hypo at least four-fold. Based on these differentially expressed lncRNAs, a clear distinction was generated by cluster analysis between control males and NOA (MA and Hypo) patients (Figure 1a). lncRNAs that changed significantly are displayed in Figure 1b. NLC1-C is marked with a red arrow. To confirm the results obtained by microarray analysis, quantitative real-time PCR analysis of normal and NOA testicular samples was performed for NLC1-C, HOTTIP, imsncRNA320 and imsncRNA292. Consistent with the microarray analysis results, expression of NLC1-C and HOTTIP was down-regulated (Figure 1c; Supplementary Figure S1a) and imsncRNA320 and imsncRNA292 were up- regulated (Supplementary Figure S1b and c) in patients with NOA (including MA and Hypo). Furthermore, *in situ* hybridisation (ISH) data showed that in seminiferous tubules with normal spermatogenesis, NLC1-C expression was predominantly found in the cytoplasm of spermatogonia and primary spermatocytes (the left panel of Figure 1d), while the expression of NLC1-C was accumulated in the nucleus of spermatogonia and primary spermatocytes in the testes of infertile men with mixed patterns of MA (the right panel of Figure 1d (marked with the black arrow)). NLC1-C, which is highly expressed in the hypothalamus and sperm,^36^ was also rich expressed in the NTERA-2 (NT2) and NCCIT cell line (Figure 1e). As shown in Figure 1f and g, NLC1-C expressed both in the cytoplasm and nucleus of NT2 cells. Since high expression of NLC1-C was observed in NT2 and NCCIT cells, the effect of NLC1-C on NT2 cell survival was evaluated. Overexpression of NLC1-C (Supplementary Figure S2a) significantly accelerated NT2 cell proliferation (Figure 1i). Conversely, knockdown of NLC1-C (Supplementary Figure S2b and c) not only decreased NT2 cells proliferation (Figure 1h), but also promoted NT2 cells apoptosis (Figure 1j and k).

### NLC1-C directly binds to Nucleolin in the nucleus

**Figure 2:**
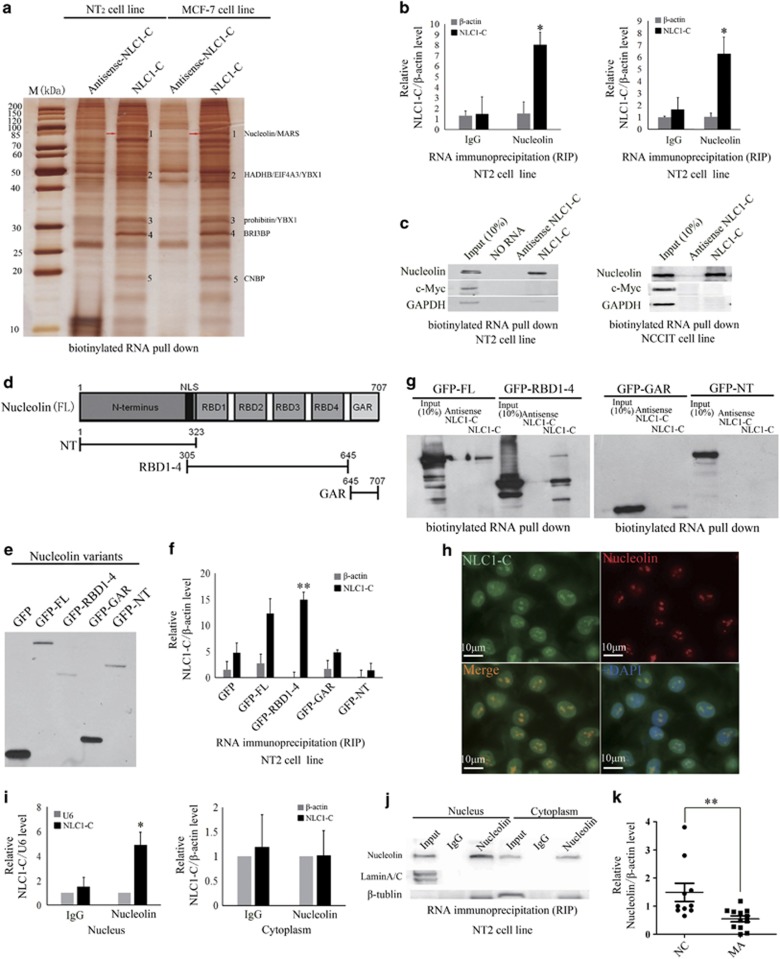
NLC1-C directly binds to Nucleolin RBD domain in the nucleus. (**a**) Identification of cellular proteins associated with NLC1-C *in vitro*. Proteins from NT2 cell extracts were pulled down with the biotinylated RNAs, subjected to SDS-PAGE, and visualised by silver staining. The bands specific to NLC1-C, as indicated by the numbers (bands 1–5), were subjected to mass spectrometry. The Nucleolin (**b** and **1**) is indicated by the red arrow. (**b**) NLC1-C interacts with Nucleolin *in vivo*. Nucleolin was immunoprecipitated from NT2 cells, NCCIT cells and co-precipitated RNAs were detected by qRT-PCR using primers for NLC1-C and *β*-actin (as negative control). IP enrichment was determined as the amount of RNA associated to the Nucleolin IP relative to IgG control. Data are from one of three independent experiments and are represented as mean±S.D. with *n*=3. (**c**) Immunoblotting analysis of the specific interaction of NLC1-C with Nucleolin. Proteins from NT2 and NCCIT cells pulled down with the biotinylated RNAs as in (**a**) were analysed by immunoblotting with a Nucleolin antibody. c-Myc and GAPDH were used as negative controls. (**d**) Schematic representation of the primary sequence of Nucleolin. Acidic stretches are in the N-terminal region; four RNA-binding domains (RBD), called RNA recognition motifs (RRM), are in the central region; and a glycine/arginine-rich domain or GAR domain is at the C-terminus. (**e**) Western blot analysis of the expression of the GFP-tagged Nucleolin constructs. The various Nucleolin constructs, as indicated, were transfected into NT2 cells for 48 h. Lysates were prepared from these cells and examined by GFP antibodies. (**f**) Elucidation of a direct Nucleolin–NLC1-C interaction to the RNA-binding domain. The various Nucleolin constructs, as indicated, were transfected into NT2 cells for 48 h. Lysates were prepared from these cells. RNAs that were co-precipitated with GFP were detected by qRT-PCR using primers for NLC1-C and *β*-actin (as negative control). IP enrichment was determined as the amount of RNA associated to the various Nucleolin domains. Data are from one of three independent experiments and are represented as mean±S.D. with *n*=3. (**g**) Immunoblotting analysis of the specific interaction of NLC1-C with the Nucleolin RBD domain. Immunoblotting analysis of the specific interaction of NLC1-C with Nucleolin. Proteins pulled down with the biotinylated RNAs as in (**a**) were analysed by immunoblotting with GFP antibody. (**h**) Co-localisation analysis: RNA FISH assay of NLC1-C combined with immunofluorescence detection of Nucleolin in NT2 cell. Scale bars, 10 *μ*m. DAPI, 4′,6-diamidino-2-phenylindole. (**i**) NLC1-C directly binds to Nucleolin in nucleus. Nucleolin was immunoprecipitated from NT2 subcellular fractions and co-precipitated RNAs were detected by qRT-PCR using primers for NLC1-C and *β*-actin (the negative control of cytoplasmic RNA) and U6 (the negative control of nuclear RNA). IP enrichment was determined as the amount of RNA associated to the Nucleolin IP relative to IgG control. Data are from one of three independent experiments and are represented as mean±S.D. with *n*=3. (**j**) Western blotting shows the amount of Nucleolin in input, IgG and immunoprecipitated Nucleolin containing mRNP complexes from both nucleus and cytoplasm fractions. Lamin A/C and *β*-tubulin were used as markers for nucleus and cytoplasm fractions, respectively (**k**). The expression of Nucleolin was down-regulated in MA patients. Quantitative real-time PCR analysis of the expression of Nucleolin in MA patients and normal control

As previous studies showed that lncRNAs function by interacting with proteins,^37, 38, 39^ we assumed that the role of NLC1-C in cell proliferation and apoptosis might be mediated by such a mechanism. To identify the proteins that are associated with NLC1-C, we next performed pull-down assays with biotinylated NLC1-C. As shown in Figure 2a, compared with antisense NLC1-C (lanes 2 and 4 from the left), there were five bands that specifically associated with NLC1-C (lanes 3 and 5 from the left). The protein bands specific to NLC1-C were then extracted, digested with trypsin, and subjected to mass spectrometry analysis. Interestingly, this analysis showed that the majority of these proteins binding to the NLC1-C transcript were associated with pre-mRNAs processing (Figure 2a). Considering NLC1-C's roles in the control of cell proliferation and apoptosis, Nucleolin (Figure 2a, marked with red arrow ), an RNA-binding protein which played a critical role in tumorigenesis and angiogenesis,^40, 41, 42^ was chosen for further studies. First, the association of NLC1-C with Nucleolin was validated by RNA immunoprecipitation (RIP). NLC1-C was clearly detected in the RNAs associated with Nucleolin, but not in the complexes associated with FLAG and GFP control *in vitro* (Supplementary Figure S3a and b) and IgG control *in vivo* (Figure 2b). RNA pull-down assays further verified the specificity of this interaction *in vivo* (Figure 2c). As shown in the Figure 2d, Nucleolin has three domains: an N-terminus containing acidic stretches (NT); four RNA-binding domains (RBD), called RNA recognition motifs (RRM), in the central region; and a glycine/arginine-rich domain, GAR domain, at the C-terminus. To establish a direct interaction between NLC1-C and Nucleolin, we used EGFP-tagged Nucleolin constructs (Figure 2e)^43^ in RIP and RNA pull-downs. As shown in the Figure 2f and g, immunoprecipitation with GFP or pulling down with biotinylated NLC1-C revealed that NLC1-C specifically binds to the RBD domain of Nucleolin and not to the GAR or NT domains. Moreover, RNA FISH followed by immunofluorescence (Figure 2h) and RIP using extracts from NT2 cells that had been fractionated into different subcellular compartments (Figure 2i and j) showed that NLC1-C co-localised with Nucleolin in the nucleus, but not the cytoplasm. Correlating with NLC1-C expression, the expression of Nucleolin was also down-regulated in MA patients compared with normal control (Figure 2k). These observations suggest that NLC1-C directly binds to the RNA-binding domain of Nucleolin in the nucleus of NT2 and NCCIT cells.

### NLC1-C is a direct target of miR-320a and miR-383

**Figure 3:**
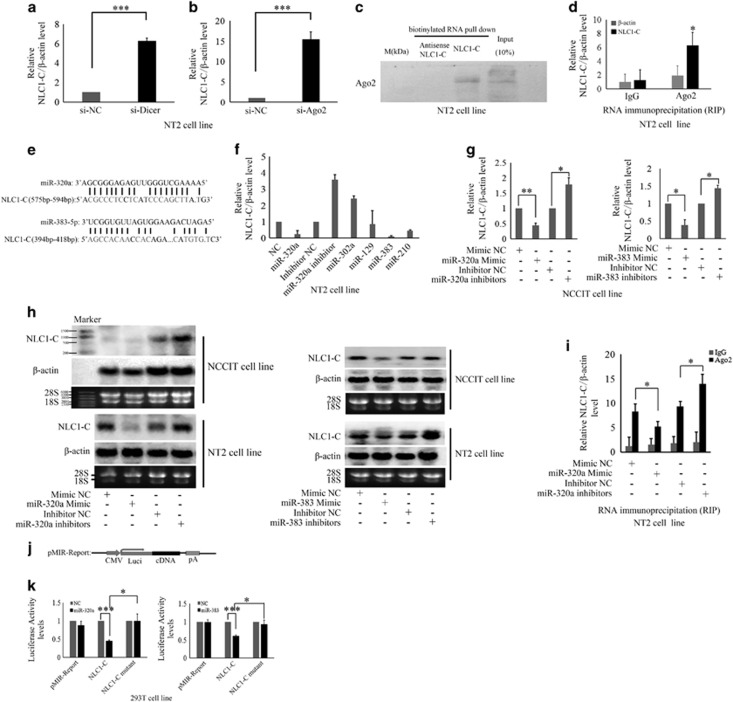
The expression of NLC1-C is inhibited by miR-320a and miR-383. (**a** and **b**) Knockdown of Dicer and Ago2 increased NLC1-C expression. NT2 cells were transfected with control siRNA (si-NC) or Dicer or Ago2 siRNAs separately. The expression of NLC1-C was detected by quantitative real-time PCR after transfection for 48 h. *β*-Actin was used as an internal reference. (**c**) Immunoblotting analysis of the interaction of NLC1-C with Ago2. Proteins from NT2 cells extracts were pulled down with the biotinylated RNAs, subjected to SDS-PAGE, and analysed by immunoblotting with Ago2 antibody. (**d**) NLC1-C interacts with Ago2 *in vivo*. Ago2 was immunoprecipitated from untreated NT2 cells and co-precipitated RNAs were detected by qRT-PCR using primers for NLC1-C and *β*-actin (as negative control). IP enrichment was determined as the amount of RNA associated in the Ago2 IP relative to IgG control. (**e**) The sequences of miR-320a and miR-383 complement with NLC1-C. (**f** and **g**) The expression of NLC1-C was repressed by miR-320a and miR-383. Quantitative real-time PCR analysis of NLC1-C expression after NT2 and NCCIT cells were transfected with indicated miRNAs for 48 h. Data are represented as mean±S.D. from three independent experiments. *P<0.05 or **P<0.01 compared with control RNA. (**h**). The expression of NLC1-C was repressed by miR-320a and miR-383. Total RNAs were collected from NT2 and NCCIT cells transfected with the indicated miRNAs individually and then resolved on a denatured agarose gel. Transcripts of different types of expressed RNAs were probed with Dig-labelled antisense NLC1-C and *β*-actin. *β*-Actin was used as an internal reference Equal amounts of total RNAs were loaded onto an agarose gel and rRNAs were used as the loading control. (**i**) miR-320a affected the interaction of Ago2 with NLC1-C in NT2 cells. Ago2 was immunoprecipitated from NT2 cells transfected with the indicated miRNAs and co-precipitated RNAs were detected by qRT-PCR using primers for NLC1-C and *β*-actin (as negative control). IP enrichment was determined as the amount of RNA associated in the Ago2 IP relative to IgG control. Data are represented as mean±S.D. from three independent experiments. *P<0.05 compared with control RNA. (**j**) The structure of pMIR-REPORT miRNA Expression Reporter Vector. (**k**) miR-320a and miR-383 repressed NLC1-C luciferase activity and the mutation rescued the luciferase activity

Previous reports showed that lncRNA communicates with mRNAs,^44, 45, 46^ pseudogenes,^47^ circular RNAs^48^ and miRNAs, acting as endogenous miRNA sponges,^49, 50^ ceRNAs^21^ or a target of miRNA.^51, 52^ To further investigate whether NLC1-C communicates with miRNAs in controlling NT2 and NCCIT cell survival, we knocked down RISC proteins (e.g., Argonaute2 (Ago2) and Dicer) (Supplementary Figure S4a and b). As shown in Figure 3a and b, silencing of Ago2 and Dicer promoted NLC1-C expression in NT2 cells, while, it repressed Nucleolin RNA and protein expression (Supplementary Figure S4c and d). Next, we examined whether Ago2 binds to NLC1-C. The association of NLC1-C with Ago2 was validated by pulling-down NLC1-C and immunoblotting for Ago2 (Figure 3c). To further confirm the interaction between NLC1-C and Ago2 *in vivo*, RIP was performed. An antibody against Ago2 was incubated with NT2 cell lysates, and co-precipitated RNAs were then analysed by real-time PCR using primers for NLC1-C or *β*-actin (negative control). As expected, we observed an enrichment of NLC1-C, but not *β*-actin, in Ago2 immunoprecipitates when compared to non-specific IgG control (Figure 3d). These data demonstrate that NLC1-C is regulated by miRNAs. We then wanted to determine which miRNAs regulated NLC1-C expression. Analysis of bioinformatics data revealed one seed site for miR-320a and miR-383, separately, in the NLC1-C RNA sequence (Figure 3e). A series of indicated miRNAs were transfected into NT2 cells and NCCIT cells. Real-time PCR analysis and northern blotting (NB) assay showed that the expression of NLC1-C was significantly inhibited by miR-320a/miR-383 mimics and was promoted by miR-320a/miR-383 inhibitors (Figure 3f and h). while, as shown in Supplementary Figure S5a and b, the expression of Nucleolin mRNA and protein level was not affected by miR-320a/miR-383 mimics and inhibitors. Moreover, overexpression of miR-320a weakened the interaction of Ago2 with NLC1-C in NT2 cells and inhibition of miR-320a increased the interaction of Ago2 with NLC1-C (Figure3i). To validate whether the NLC1-C gene is a direct target of miR-320a and miR-383, we constructed luciferase reporter constructs containing either the wild-type (WT) full-length NLC1-C RNA or the mutant forms of the seed sites into the pMIR-Report vector (Figure 3j). Co-transfection of the miR-320a or miR-383 mimic and the reporters into 293 T cells resulted in an ~50% decrease in luciferase activity (Figure 3k), whereas mutation of the seed sequences abolished the silencing effects of miR-320a or miR-383 (the right panel of Figure 3k). Taken together, these results demonstrate that NLC1-C is a direct target of miR-320a and miR-383.

### NLC1-C represses the expression of miR-320a and miR-383 directly binds Nucleolin in the nucleus

**Figure 4:**
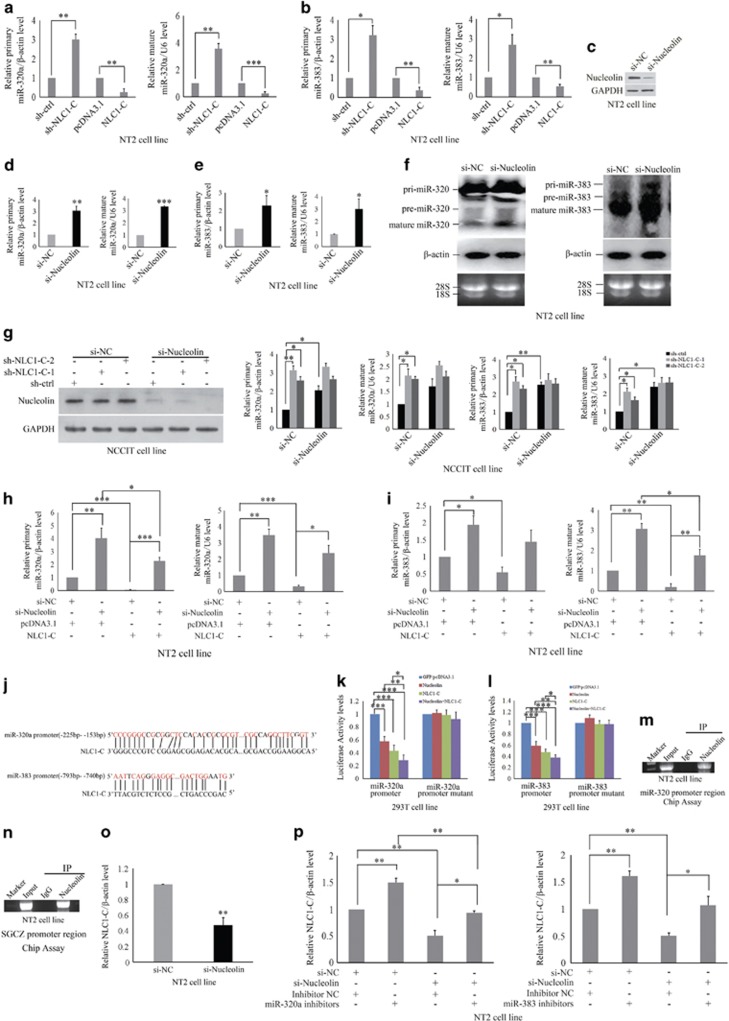
NLC1-C represses miR-320a and miR-383 transcription by binding to Nucleolin. (**a** and **b**) NLC1-C inhibited primary and mature miR-320a/383 expression. NT2 cells expressing under infection of control shRNA or NLC1-C shRNA lentivirus particles or transfection of pcDNA3.1 or NLC1-C-pcDNA3.1(NLC1-C ) plasmids were cultured for 48 h. Total RNA was then subjected to real-time RT-PCR analysis. (**c**) The efficacy of Nucleolin siRNA was evaluated by western blotting after 48 h transfection with either siRNA negative control (si-NC) or Nucleolin siRNA (si-Nucleolin). (**d**–**f**) Knockdown of Nucleolin promoted primary and mature miR-320a/miR-383 expression. NT2 cells were transfected with 5 nM si-NC/si-Nucleolin, the levels of miR-320a and miR-383 were determined after 48 h by real-time PCR (**d** and **e**) and northern blot (**f**). (**g**) Nucleolin and NLC1-C co-regulated the expression of primary and mature miR-320a/miR-383. The expression levels of Nucleolin and the primary and mature miR-320a/miR-383 were analysed after transfection with 5 nM si-NC/si-Nucleolin and infection with sh-ctrl, sh-NLC1-C-1 and sh-NLC1-C-2 into NCCIT cells for 48 h by western blotting and real-time PCR. (**h** and **i**) Nucleolin and NLC1-C co-regulated the expression of primary and mature miR-320a/miR-383. The expression levels of primary and mature miR-320a/miR-383 were analysed after co-transfection with 5 nM si-NC/si-Nucleolin and 4 *μ*g pcDNA3.1/NLC1-C into NT2 cells for 48 h by real-time PCR. (**j**) The sequences of NLC1-C complement with miR-320a and miR-383 promoter. (**k** and **l**) NLC1-C and Nucleolin inhibited miR-320a/miR-383 expression by binding to their promoters. Luciferase reporters containing either miR-320 promoter/mutant or miR-383 promoter/mutant were co-transfected with the indicated plasmids into 293 T cells. Luciferase activity was measured after 30 h. (**m** and **n**) ChIP analysis reveals *in vivo* binding of Nucleolin to the miR-320/ human SGCZ (miR-383) promoter. ChIP assays were performed on NT2 cells extracts using antibodies against IgG and Nucleolin, followed by PCR amplification with primers sequences corresponding to the miR-320a/human SGCZ (miR-383) promoter region. (**o**) Knockdown of Nucleolin repressed NLC1-C expression. NT2 cells were transfected with either siRNA negative control (si-NC) or Nucleolin siRNA (si-Nucleolin) for 48 h. Total RNA was then subjected to real-time PCR analysis. (**p**) miR-320a/383 inhibitors rescued Nucleolin siRNA-induced NLC1-C expression repression. Real-time PCR analysis of NLC1-C expression after NT2 cells were co-transfected with 5 nM si-NC/si-Nucleolin and 120 nM NC/miR-320a/miR-383 inhibitors into NT2 cells for 48 h. Data are presented as means±S.E.M. for at least three independent experiments. *P<0.05, **P<0.01, ***P<0.001, compared with negative controls

Since NLC1-C is a direct target of miR-320a and miR-383, we wanted to determine whether NLC1-C can regulate the expression of miR-320a and miR-383. We used lentivirus-mediated RNA interference (RNAi) to knockdown NLC1-C. As shown in Figure 4a and b, silencing NLC1-C increased primary and mature miR-320a and miR-383 expression, whereas overexpression of NLC1-C decreased their expression. Several studies have demonstrated that Nucleolin affects the expression of a specific set of miRNAs through a post-transcriptional mechanism.^42, 53^ We then sought to determine whether Nucleolin post-transcriptionally regulates the expression of miR-320a and miR-383. After 48 h of Nucleolin knockdown (Figure 4c), the expression of primary and mature miR-320a/miR-383 was increased (Figure 4d–f), similar to NLC1-C knockdown. Next, we examined whether Nucleolin and NLC1-C co-regulate miR-320a and miR-383 expression. As Figure 4g shown, Knockdown of NLC1-C (marked with sh-NLC1-C-1 and sh-NLC1-C-2) or Nucleolin (si-Nucleolin), respectively, increased the expression of miR-320a and miR-383. However, NLC1-C shRNA could not increase more in the presence of Nucleolin siRNA (the last two columns in the right half panel of each Figure 4g histogram) compared with NLC1-C shRNA single (the last two columns in the left half panel of each Figure 4g histogram). Overexpression of NLC1-C (the fourth column from the left in each Figure 4h and i) could partly repress miR-320a/383 expression in cells with si-Nucleolin (the second column from the left in each Figure 4h and i). Analysis of bioinformatics data revealed that there is one binding site of NLC1-C on the miR-320 and SGCZ (miR-383) promoter (Figure 4j). To verify that these potential NLC1-C/Nucleolin-binding sites were important for the transcriptional activity of miR-320 and miR-383, expression vectors encoding Nucleolin and/or NLC1-C were transiently co-transfected with miR-320 or SGCZ (miR-383) promoter reporter plasmids into 293 T cells. Overexpression of Nucleolin and NLC1-C decreased the activity of the miR-320/SGCZ (miR-383) promoter region (−1050/+50 bp), which contains a putative NLC1-C binding site located at −225  to −153 bp (miR-320 promoter) or −793  to −740 bp (SGCZ (miR-383) promoter), by about 50 and 50%, respectively (Figure 4k and l). Concurrent forced expression of Nucleolin and NLC1-C further reduced luciferase expression (Figure 4k and l). Conversely, Mutation of putative NLC1-C binding site in the miR-320/SGCZ (miR-383) promoter region recued the luciferase activity repression induced by Nucleolin and NLC1-C (Figure 4k and l). These results indicate that the miR-320 and SGCZ promoter region might contain a critical Nucleolin-responsive regulatory element. Chip IP (ChIP) assay with NT2 cells further revealed the binding of Nucleolin to the promoter elements in the 5′-flanking region of the miR-320 and SGCZ gene (Figure 4m and n). Antibodies to normal IgG failed to immunoprecipitate miR-320 and SGCZ promoter (Figure 4m and n). These findings demonstrate that Nucleolin and NLC1-C repress the expression of miR-320a/miR-383 at the level of transcription and not through post-transcriptional regulation. Furthermore, some studies have been shown that the RBD domain of Nucleolin interacts with RNA and alters its stability.^54, 55^ As shown in Figure 4o, knockdown of Nucleolin decreased the expression of NLC1-C. Since NLC1-C was a direct target of miR-320a and miR-383 and Nucleolin promoted NLC1-C expression, we investigated whether miR-320a and miR-383 mediated the increase induced by Nucleolin on NLC1-C expression. We subsequently analysed the expression of NLC1-C in NT2 cells co-transfected with Nucleolin siRNA and miR-320a or miR-383 inhibitors. As shown in Figure 4p, the inhibitors of miR-320a or miR-383 were sufficient to rescue the decrease induced by Nucleolin depletion on NLC1-C expression. These results demonstrate that Nucleolin binding to NLC1-C co-regulates miR-320a and miR-383 transcription mediating the increase of NLC1-C expression induced by Nucleolin.

### The accumulation of NLC1-C in the nucleus represses both miR-320a and miR-383 transcription by binding to Nucleolin is associated with male infertility

**Figure 5:**
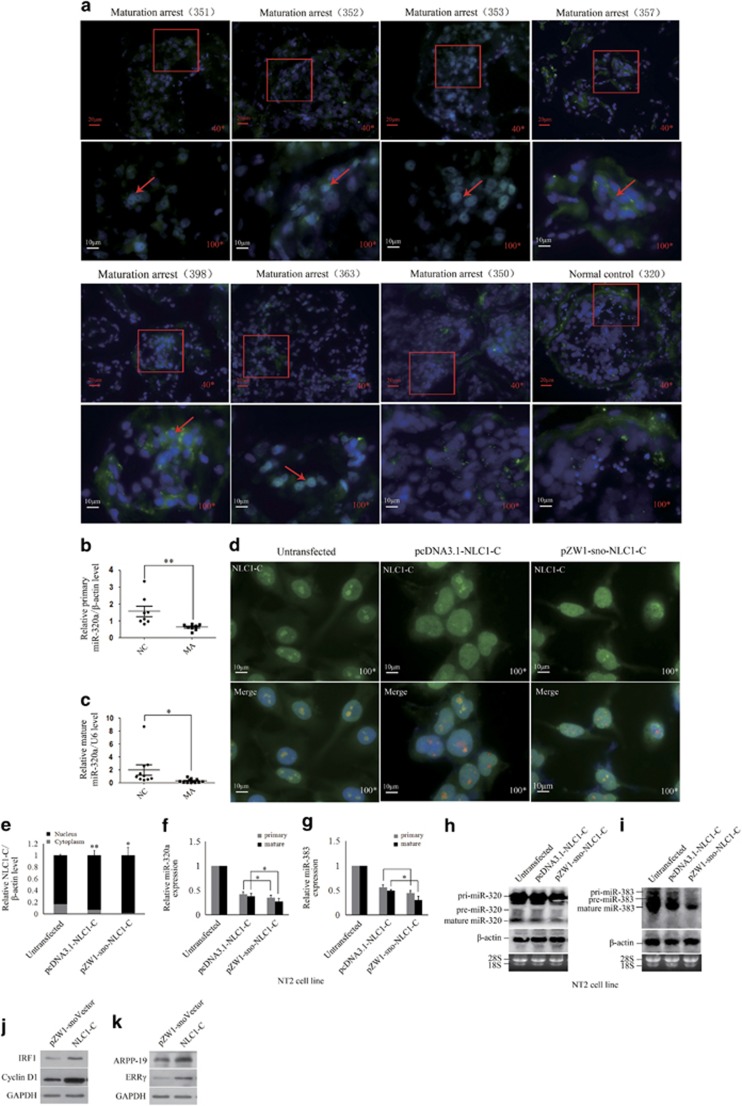
The accumulation of NLC1-C in the nucleus represses miR-320a and miR-383 transcript by binding to Nucleolin and is associated with male infertility. (**a**) NLC1-C accumulated in the nucleus of spermatogonia and primary spermatocytes in the testes of infertile men with mixed patterns of MA compared with normal controls. RNA *in situ* hybridisation was performed with DIG-labelled NLC1-C in the testes of infertile men with mixed patterns of MA and normal controls. Top: Representative images are shown for × 40 magnification. Scale bars 20 *μ*m. The images in the red box and red arrow heads represent the nuclear signal of NLC1-C in the nucleus of spermatogonia and primary spermatocytes. All nuclei were counterstained with DAPI. (**b** and **c**) The primary and mature miR-320a expression was down-regulated in MA patients. Quantitative real-time PCR analysis of the primary and mature miR-320a expression in normal testes and MA patients testes. (**d** and **e**) NT2 cells were transfected with indicated plasmids, the accumulation of NLC1-C in the nucleus was increased and the signal of NLC1-C binding to Nucleolin was enhanced. NT2 cells were transfected individually with the indicated plasmids. Fractionated nuclear and cytoplasmic RNAs were collected 48 h after transfection and then subjected to quantitative real-time PCR assay. Transcripts of NLC1-C were probed with DIG-labelled NLC1-C combined with immunofluorescence detection of Nucleolin in NT2 cells. (**f** and **g**) The expression of primary and mature miR-320a/383 was inhibited more significantly when NT2 cells were transfected with pZW1-sno-NLC1-C plasmids compared with pcDNA3.1-NLC1-C. NT2 cells were transfected individually with the indicated plasmids for 48 h, and then subjected to quantitative real-time PCR assay. Data are presented as means±S.E.M. for at least three independent experiments. *P<0.05, **P<0.01, ***P<0.001, compared with negative controls. (**h** and **i**) The expression of primary and mature miR-320a/383 was inhibited more significantly when NT2 cells were transfected with pZW1-sno-NLC1-C plasmids compared with pcDNA3.1-NLC1-C. NT2 cells were transfected individually with the indicated plasmids for 48 h, and then subjected to northern blotting assay. (**j** and **k**) The expression of miR-383 targets IRF1 and Cyclin D1 (**j**) and miR-320a targets ARPP-19 and ERR*γ* (**k**) was promoted by pZW1-sno-NLC1-C. Western blot analysis of the expression of IRF1, Cyclin D1, ARPP-19 and ERR*γ* after NT2 cells were transfected individually with the indicated plasmids for 48 h

The expression and the location of NLC1-C in the testes of infertile men with mixed patterns of MA were determined by RNA ISH. In seminiferous tubules with normal spermatogenesis, NLC1-C expression was predominantly found in the cytoplasm of spermatogonia and primary spermatocytes (the last section of Figure 5a). However, the expression of NLC1-C accumulated in the nucleus of spermatogonia and primary spermatocytes in the testes of infertile men with mixed patterns of MA (Figure 5a). Our previous results found that hyperactive proliferation of germ cells in patients with mixed patterns of MA and down-regulation of primary and mature miR-383 was associated with enhanced proliferative activity of germ cells in MA patients.^8^ In this study, we found that the expression of primary and mature miR-320a was also down-regulated in patients with mixed patterns of MA (Figure 5b and c). As described in this study, NLC1-C binding to Nucleolin co-regulates miR-320a and miR-383 transcription. To validate whether the accumulation of NLC1-C in the nucleus represses both miR-320a and miR-383 transcripts resulting in hyperactive proliferation of germ cells associated with male infertility, we transfected NT2 cells with pcDNA3.1-NLC1-C or pZW1-sno-NLC1-C plasmid, which stably expresses a nuclear NLC1-C. Forty-eight hours after transfection, RNA ISH followed by immunofluorescence or real-time PCR was performed. As shown in Figure 5d and e, after NT2 cells were transfected with pcDNA3.1-NLC1-C or pZW1-sno-NLC1-C plasmids, the accumulation of NLC1-C in the nucleus was increased and the signal of NLC1-C binding to Nucleolin was significantly enhanced. In addition, we observed that the expression of primary and mature miR-320a/383 was inhibited more significantly when NT2 cells were transfected with pZW1-sno-NLC1-C compared with pcDNA3.1-NLC1-C (Figure 5f–i). The expression of miR-383 targets IRF1 and Cyclin D1^8, 56^ (Figure 5j) and miR-320a targets ARPP-19 and ERR*γ*^57^ (Figure 5k) was promoted by pZW1-sno-NLC1-C. These observations and our previous results ^8, 56^ confirmed that the accumulation of NLC1-C in the nucleus of spermatogonia and primary spermatocytes represses miR-320a and miR-383 transcripts resulting in hyperactive proliferation of germ cells leading to male infertility.

### miR-320a and miR-383 mediate the proliferation and apoptosis of NT2 cells induced by NLC1-C depletion

**Figure 6:**
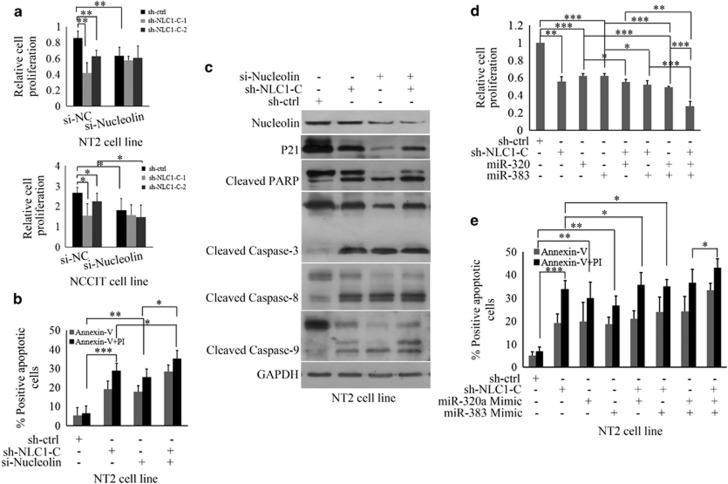
miR-320a and miR-383 mediates the proliferation and apoptosis of NT2 cells induced by NLC1-C depletion. (**a**) Silencing of Nucleolin enhanced sh-NLC1-C-induced suppression of proliferation. Cell viability was analysed after co-transfection with 5 nM si-Nucleolin and lentivirus-mediated RNA interference (RNAi) to knockdown NLC1-C in NT2 cells by CCK-8. (**b**) Silencing Nucleolin increased NT2 cells apoptosis induced by NLC1-C depletion. Apoptotic rates were determined using flow cytometry after lentivirus-mediated RNA interference (RNAi) to knockdown NLC1-C and transfection with 5 nM si-Nucleolin in NT2 cells for 48 h. Data are presented as means±S.E.M. for at least three independent experiments. *P<0.05, **P<0.01, ***P<0.001, compared with negative controls. (**c**) p21 expression was repressed after NLC1-C and Nucleolin depletion and the activated caspase-3, -8, -9 and cleaved PARP were more obvious in the combination of NLC1-C and Nucleolin depletion than single NLC1-C or Nucleolin silencing. Western blot analysis of indicated protein expression in NT2 cells after transfecting with 5 nM Nucleolin siRNA and lentivirus-mediated RNAi to knockdown NLC1-C for 48 h. (**d**) Silencing of NLC1-C enhanced miR-320/383-induced proliferation suppression. Cell viability was measured after lentivirus-mediated RNAi to knockdown NLC1-C and transfection with 80 nM miR-320/383 mimics into NT2 cells by the CCK-8 assay. (**e**) Silencing of NLC1-C enhanced miR-320/383-induced NT2 cells apoptosis. Apoptotic rates were determined using flow cytometry after lentivirus mediated RNAi to knockdown NLC1-C and transfection with 80 nM miR-320/383 mimics into NT2 cells for 48 h. Data are presented as means±S.E.M. for at least three independent experiments. *P<0.05, **P<0.01, ***P<0.001, compared with negative controls

Since knockdown of NLC1-C inhibited NT2 cell proliferation, we then sought to determine whether depletion of Nucleolin enhances NLC1-C siRNA-mediated repression of NT2 cell and NCCIT cell proliferation. Simultaneous knockdown of NLC1-C and Nucleolin substantially decreased NT2 cell and NCCIT cell growth when compared with NLC1-C or Nucleolin depletion (Figure 6a). Additionally, we found that silencing Nucleolin increased NT2 cell apoptosis induced by NLC1-C depletion (Figure 6b). We were then interested in identifying a major gene responsible for NLC1-C shRNA and Nucleolin siRNA-mediated apoptosis in NT2 cells. Protein expression of Nucleolin, p21, PARP, caspase-3, caspase-8 and caspase-9 were analysed after NLC1-C and Nucleolin depletion. As shown in Figure 6c, p21 expression was repressed after NLC1-C and Nucleolin depletion, and activated caspase-3, -8, -9 and cleaved PARP were more obvious in the combination of NLC1-C and Nucleolin depletion than single NLC1-C or Nucleolin silencing. We next examined the effects of miR-320a/miR-383 on NT2 cell proliferation and apoptosis induced by NLC1-C. Our results revealed that co-transfection of any two of miR-320a mimic, miR-383 mimic and NLC1-C shRNA into NT2 cells resulted in more significant decrease in cell proliferation than transfection with any one of them (Figure 6d). NT2 cell proliferation was inhibited the most significantly when transfected in combination with the miR-320a mimic, miR-383 mimic, and NLC1-C shRNA (Figure 6d). Similarly, miR-320a and miR-383 enhanced apoptosis of NT2 cells induced by NLC1-C depletion (Figure 6e). These findings suggest that miR-320a and miR-383 mediate the proliferation and apoptosis of NT2 cells induced by NLC1-C depletion.

## Discussion

**Figure 7:**
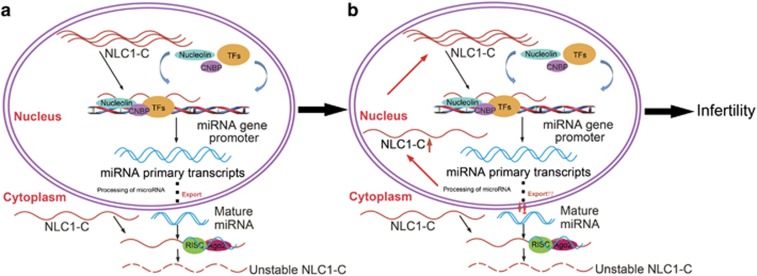
Proposed model for the dysregulation of NLC1-C in the process of spermatogenesis resulting in male infertility. (**a**) In the normal male testis, NLC1-C inhibits miR-320a and miR-383 transcripts by binding to Nucleolin in the nucleus. When NLC1-C and precursor miR-320a/383 are exported to the cytoplasm, precursor miR-320a/383 are processed into mature forms that target NLC1-C to regulate spermatogenesis. (**b**) NLC1-C is down-regulated in the cytoplasm and meanwhile it accumulates in the nucleus of spermatogonia and primary spermatocytes repressing miR-320a and miR-383 transcripts, leading to hyperactive proliferation of spermatogonia and primary spermatocytes by binding to Nucleolin, ultimately resulting in male infertility

Spermatogenesis is a complex and highly coordinated process that depends not only on the proliferative activity of spermatogonia but also the loss of germ cells during meiosis and spermiogenesis.^58^ In this study, we found that in the normal male testes, NLC1-C inhibited miR-320a and miR-383 transcripts by binding to Nucleolin in the nucleus. When NLC1-C and precursor miR-320a/383 were exported to the cytoplasm, precursor miR-320a/383 were processed into mature forms targeting NLC1-C to regulate cell survival (Figure 7a). However, when NLC1-C was down-regulated in the cytoplasm, it accumulated in the nucleus of spermatogonia and primary spermatocytes, repressing miR-320a and miR-383 transcripts and leading to hyperactive proliferation of spermatogonia and primary spermatocytes by binding to Nucleolin, ultimately resulting in male infertility (Figure 7b). In addition, the NLC1-C/Nucleolin-miR-320a/miR-383-NLC1-C pathways are identified in the regulation of NT2 cell survival. We provide, for the first time, mechanistic insights into the role of NLC1-C in spermatogenesis and in NT2 cell proliferation.

Recent studies have shown that NLC1-C is highly expressed in human whole brain and sperm,^36^ specifically expressed in the white matter of the human frontal lobe.^59^ In this study, the altered lncRNA expression profiles in the testes of patients with MA and normal controls were performed using microarray technology. NLC1-C was down-regulated in the testes of MA patients. However, NLC1-C was highly expressed in the cytoplasm of spermatogonia and primary spermatocytes of normal human testes. In addition, NLC1-C inhibited miR-320a and miR-383 transcripts *in vitro* and the expression of NLC1-C was accumulated in the nucleus of spermatogonia and primary spermatocytes in the testes of infertile men with mixed patterns of MA. Combined with the observation that the expression of primary and mature miR-320a and miR-383 was down-regulated in MA patients and the hyperactive proliferation of germ cells in patients with mixed patterns of MA,^8^ NLC1-C could contribute to spermatogenic arrest caused by the dysregulation of germ cell proliferation by accumulating in the nucleus of spermatogonia and primary spermatocytes, repressing miR-320a and miR-383 transcripts, resulting in hyperactive proliferation of germ cells. Furthermore, NLC1-C was highly expressed in the nucleus of NT2 and NCCIT (testicular embryonal carcinoma) cells, controlling cell proliferation and apoptosis. These observations may be consistent with the reports that infertile men are at a increased risk of testicular cancer.^60, 61, 62^

In this study, we found that the expression of NLC1-C accumulated in the nucleus of spermatogonia and primary spermatocytes in the testes of infertile men with mixed patterns of MA compared with normal control. The mechanism of the accumulation of NLC1-C in the nucleus requires further investigation. The starbase (http://starbase.sysu.edu.cn/) predicted that NLC1-C binds to UPF1, eIF4AIII, PTB, FUS and U2AF65. These proteins, which are associated with pre-mRNAs in the nucleus and appear to influence pre-mRNA processing, mRNA nuclear export and mRNA surveillance,^63, 64, 65^ may be dysregulated, resulting in NLC1-C nuclear export being hindered or NLC1-C located in the cytoplasm being transported into the nucleus again, or in some RNA-binding protein interaction with NLC1-C and its retention in the nucleus.^66^

Nucleolin, a RNA-binding protein, mainly localised in the nucleolus, participates extensively in RNA regulatory mechanisms, including transcription, ribosome assembly, mRNA stability and translation, and miRNA processing.^41, 53^ In our report, NLC1-C interacts with Nucleolin and CNBP (Supplementary Figure S3c) regulating miRNA transcripts involved in spermatogenesis.

In summary, we demonstrate that NLC1-C binding to Nucleolin functions as a positive regulator of cell proliferation by directly repressing miR-320a and miR-383 transcripts. Dysregulation of NLC1-C/Nucleolin-miR-320a/miR-383-NLC1-C pathways is associated with spermatogeneic arrest. The mechanistic studies regarding the role of lncRNAs in spermatogenesis will provide new clues in treating male infertility or, on the opposite side, designing new male contraceptives.

## Materials and methods

### Human testicular samples

Testicular samples used for lncRNA microarray were obtained from the First Affiliated Hospital of Anhui Medical University (Hefei, China). Five patients (aged 24–36 years) with NOA (3 MA and 2 Hypo) and three control subjects (aged 48–60 years) undergoing orchiectomy for prostate carcinoma were recruited for this study. The testicular histology of the NOA patients showed a global early MA pattern. The control samples were analysed from urology patients who had no history of meiotic defects or infertility and histological examination showed normal spermatogenesis.

Testicular biopsy specimens used for ISH and RNA FISH from 35 patients (aged 20–35 years) with NOA (20 MA and 15 Hypo) and from 11 control subjects (aged 20–44 years) were obtained from the First Affiliated Hospital of Anhui Medical University (Hefei, China) and Renji Hospital (Shanghai, China). Each testicular specimen was divided into several portions for different experiments. The conditions used for RNA FISH are summarised in Table 1. According to standard pathological criteria, 20 patients with a mixed pattern of MA and 15 patients with Hypo were recruited for this study. A mixed pattern of MA is characterised by various spermatogenic impairments in adjacent seminiferous tubules showing both Sertoli-cell-only and spermatogenic arrest at the level of spermatogonia or primary spermatocytes. As normal controls, the study population consists of obstructive azoospermia with normal spermatogenesis. All the experiments on live vertebrates were performed in accordance with the relevant guidelines and regulations. All patients gave informed consent, and this study received ethical approval from the Institutional Review Boards of International Peace Maternity & Child Health Hospital, Renji Hospital, School of Medicine, Shanghai Jiaotong University and the Anhui Medical University.

### LncRNA microarray analysis

LncRNA expression profiles of testicular tissues from NOA (including MA and Hypo) patients and controls were generated by applying the Agilent Array platform (CapitalBio, Beijing, China). All procedures were carried out according to the manufacturer's standard protocols. Briefly, total RNA was extracted from each sample of testicular tissues using Trizol (Invitrogen, Carlsbad, CA, USA) and purified by NucleoSpin RNA clean-up Isolation Kit (MACHEREY-NAGEL, Deutschland Hamburg, Germany) according to the manufacturer's instructions. With the total RNA as a template, CbcScript enzyme synthesised the 1st-strand cDNA utilising the T7 Oligo (dT) primer and then RNase H digested the RNA of RNA–DNA. Using the digested RNA as a primer, DNA polymerase synthesised the 2st-strand cDNA. And then T7 Enzyme Mix synthesised cRNA using the cDNAs as a template, which was transcribed into cDNA by CbcScriptllenzyme along the Random Primer. Last, the cDNAs were labelled with Cy3-dCTP (GE Healthcare Cat. No. PA53021) by KLENOW enzyme and then purified by NucleoSpinExtract II Kit (MN). The labelled cDNAs were hybridised onto the human LncRNA Array v2.0 (4*180 K, Arraystar, Shanghai, china). Microarrays with labelled samples were hybridised at 45 °C for overnight using a heat-shrunk hybridisation bag (Phalanx Hybridization Assembly, Phalanx Biotech, Taiwan, China). After having washed the slides, the arrays were scanned by an Agilent G2565CA Microarray Scanner (Agilent Technlogies, Palo Alto, CA, USA), and the acquired array images were analysed by Agilent Feature Extraction software (version 11.0.1.1). Quantile normalisation and subsequent data processing were performed using the GeneSpring GX v11.5.1 software package (Agilent Technologies). After quantile normalisation of the raw data, lncRNAs were all detected in each group; three samples having flags in Present or Marginal (All Targets Value) were chosen for further data analysis. Differentially expressed lncRNAs with statistical significance between two groups of testis were identified by Volcano Plot filtering with a threshold of fold changes >2 and *P*-values <0.05. Finally, Hierarchical clustering was performed to show the distinguishable lncRNAs expression patterns among the testicular tissues.

### Cell culture; transfection of siRNAs, miRNAs and plasmids; and Lentivirus infection

Both NTERA-2 (NT2) and NCCIT cells that derived from human embryonal carcinomas are testicular germ cell tumours. NT2 and HEK293 T cells were grown in Dulbecco's modified Eagle's medium (DMEM) supplemented with 10% fetal bovine serum (FBS) (Life Technologies Inc., Grand Island, NY, USA) and 1% antibiotics (100 U/ml penicillin and 100 mg/ml streptomycin; Life Technologies Inc.) and cultured at 37 °C in 5% CO_2_. NCCIT cells were grown in RPMI-1640 medium supplemented with 10% fetal bovine serum (Life Technologies Inc.) and 1% antibiotics (100 U/ml penicillin and 100 mg/ml streptomycin; Life Technologies Inc.) and cultured at 37 °C in 5% CO_2_. NCCIT and 293 T cells were transfected using Lipofectamine 2000 Reagent (Invitrogen). Lipofectamine RNAiMAX (Invitrogen) and Fugene HD (Roche, Basel, Switzerland) were used to transfect oligonucleotides and plasmids into NT2 cells, respectively. The transfection procedure was performed according to the manufacturers' instructions. To generate lentivirus expressing either NLC1-C or control shRNAs, HEK293T cells were grown on a 6-cm dish and transfected with 2 *μ*g of PLKO.1 NLC1-C shRNA or control vector, 2 *μ*g of pREV, 2 *μ*g of pGag/Pol and 1 *μ*g of pVSVG. Twenty-four hours after transfection, cells were cultured in DMEM media containing 20% FBS (4 ml) for an additional 24 h. The culture media containing lentivirus particles was collected by centrifugation at 1000 × *g* for 5 min and used to infect NT2 and NCCIT cells. NT2 and NCCIT cells were seeded at 60–70% confluency and infected with lentivirus culture (500 *μ*l for one well of a six-well plate) in the presence of 8 *μ*g/ml polybrene. Twenty-four hours later, the media was changed to fresh media supplemented with puromycin and incubated for an additional 24 h.

### Western blot analysis

Whole-cell lysates were prepared in RIPA buffer, the composition of which was described in J Lian *et al.*,^8^ separated by SDS-polyacrylamide gel electrophoresis (SDS-PAGE), transferred onto Hybond enhanced chemiluminescence (ECL) Nitrocellulose membrane (Amersham Biosciences, Freiburg, Germany), immunoblotted with antibodies and visualised by ECL (Kodak, Rochester, NY, USA). Protein levels were normalised to GAPDH. The information regarding the antibodies is provided in Supplementary Table S4.

### Immunoprecipitation assays

Native RIP was carried out with the EZ-Magna RIP Kit (Millipore, Bedford, MA, USA) according to the manufacturer's protocol using 20 *μ*g of rabbit anti-Nucleolin antibody (Abcam, Cambridge, MA, USA, ab70493) or rabbit IgG. The co-precipitated RNAs were extracted using TRIzol (Invitrogen) and detected by real-time PCR. Proteins isolated from the beads were detected by immunoblotting.

### Real-time PCR assay

Total RNA from NT2 cells was extracted using TRIzol (Invitrogen). cDNA was synthesised from 500 ng of purified RNA using the PrimeScript RT reagent kit (TaKaRa Bio Inc., Otsu, Japan) according to the manufacturer's instructions. Real-time PCR was performed in an Applied Biosystems StepOne real-time PCR system using the SYBR Premix Ex Taq II Kit (Takara Bio Inc.). Each sample was analysed in triplicate and the experiment was repeated three times. Gene-specific qPCR primer pairs are provided in Supplementary Table 1.

### RNA Pull-down assay

The NLC1-C cDNA was cloned into the pSPT18/19 vector. Biotin-labelled RNAs were transcribed *in vitro* by SP6 RNA polymerase (Roche) and purified using the MEGAscript Kit (Ambion). Cells were harvested and resuspended in freshly prepared binding buffer (10 mM HEPES pH 7.0, 50 mM KCl, 10% glycerol, 1 mM EDTA, 1 mM DTT, 0.5% Triton X-100) supplemented with tRNA (0.1 *μ*g/*μ*l), heparin (0.5 *μ*g/*μ*l) and RNasin (1 U). Biotin-labelled RNAs (3 *μ*g) were mixed with 200 *μ*g of whole-cell lysate and then mixed with pre-washed streptavidin-coupled Dynabeads (Invitrogen) for 1 h at RT. The beads were washed with ice cold binding buffer five times and then boiled in 2 × Laemmli loading buffer. The retrieved proteins were subjected to SDS-PAGE and further visualised by silver staining or immunoblotting. Protein bands were excised and identified by in-gel trypsin digestion followed by mass spectrometry analysis.

### Northern blot

For NLC1-C, *β*-actin, miR-320, miR-383 NB, total RNAs collected from transfected cells were resolved on 1.5% denatured agarose gels and Northern blots were carried out according to the manufacturer's protocol (DIG Northern Starter Kit, Roche). Digoxigenin (DIG)-labelled antisense of NLC1-C and *β*-actin probes were made using either SP6 or T7 RNA polymerases by *in vitro* transcription with the DIG Northern Starter Kit (Roche) and 5'-end DIG-labelled LNA modified DNA oligonucleotides (LNAs) complementary to the mature miR-320 and miR-383 were supplied by Exiqon A/S (Vedbaek, Denmark). The probes sequences of miR-320 and miR-383 are listed in Supplementary Table S3.

### ChIP assay

ChIP assay was performed using a ChIP kit (Millipore) according to the manufacturer's instructions, as described previously.^67^ Briefly, chromatin was immunoprecipitated with normal mouse IgG anti-Nucleolin antibodies. The Nucleolin-binding sites in the miR-320 promoter region and the sarcoglycan zeta (SGCZ) promoter region were amplified by PCR using primers shown in Supplementary Table S2. The amplification products were resolved by 2% agarose gel electrophoresis and visualised by SYBR Green II staining.

### Subcellular fractionation

NT2 cells were fractioned using the NE-PER Nuclear and Cytoplasmic Extraction Reagents (Thermo, Shanghai, China; 78835), according to the manufacturer's protocol. TRIzol was added to the cytoplasmic and nuclear fractions. RNA isolation and qRT-PCR were performed as described in the real-time PCR assay section using primers for NLC1-C or U6 (the negative control for nuclear RNA) or *β*-actin (the negative control for cytoplasmic RNA). To prevent amplification from possible genomic DNA contamination, the RNA was treated with DNase before being reverse transcribed.

### Cell proliferation assay

Cell proliferation was measured using the Cell Counting Kit-8 (CCK-8) (Dojindo Laboratories, Kumamoto, Japan). NT2 cells were plated at 3–5 × 10^3^ cells/well in 96-well plates with five replicate wells for each condition. Forty-eight hours after transfection, 10 *μ*l of CCK-8 solution was added to the media and then incubated at 37 °C for 2 h. Cell numbers were measured by measuring the absorbance at 450 nm using a 96-well format plate reader (ELx800 Universal Microplate Reader; Biotek Instrument Inc., Highland Park, VT, USA).

### Flow cytometric analysis

For cell apoptosis analysis, NT2 cells were seeded in six-well plates at 70–80% confluency and transfected with either 80 nM indicated siRNAs or 100 nM miR-320a/383 mimic. Cells were harvested 48 h after transfection, stained using the Annexin V FITC Apoptosis Detection Kit (Beijing Biosea Biotechnology Co., Ltd, Beijing, China) and PI staining solution, and analysed using a FACScalibur flow cytometer (BD Biosciences, San Jose, CA, USA). Data analysis was performed using WinMDI 2.9 software (The Scripps Research Institute, La Jolla, CA, USA).

### Luciferase reporter assay

To confirm that NLC1-C was the direct target of miR-320a and miR-383, 293 T cells were co-transfected with 200 ng pMIR-REPORT/Mutation plasmids, 20 ng of the transfection control Renilla vector and 80 pmol miR-320a/383 mimics/negative control for 30 h.

In the promoter activity assay, 293 T cells were co-transfected with 20 pmol Nucleolin siRNA/NLC1-C siRNA and 400 ng pGL3-miR-320a/miR-383 promoter or 400 ng pGL3-miR-320a/miR-383 promoter mutation plasmids in 24-well plates with three replicate wells for each condition.

Luciferase activity was measured 30 h after transfection using the Dual Luciferase Reporter Assay System (Promega, Madison, WI, USA). Renilla luciferase activity was normalised to Firefly luciferase activity for each transfected well. All experiments were performed in triplicate.

### RNA ISH and immunofluorescence microscopy

ISH was performed on 10 *μ*m frozen tissue sections using DIG-labelled RNAs probes (sense and antisense strands) that were transcribed *in vitro* by SP6 RNA polymerase (Roche) and purified using the MEGAscript Kit (Ambion), according to the protocol provided by the manufacturer. The probe primers are listed in Supplementary Table S3. A detailed protocol for ISH has been described previously.^8^ Sequential RNA/DNA FISH experiments were carried out. To detect the subcellular distribution of NLC1-C and the co-localisation of NLC1-C and Nucleolin in cells and tissues, RNA ISH and immunofluorescence were carried out as described in Yin *et al.*,^68^ with slight modifications. For RNA ISH, cells were denatured and then hybridised with denatured RNA probes labelled with DIG overnight. After hybridisation and washes, the sections were incubated overnight at 4 °C with 10% sheep serum containing fluorescently labelled anti-DIG. After washing three times for 5 min in PBS solution at room temperature, the sections were stained with 1 mg/ml DAPI for 10 min and then washed three times for 3 min. For co-localisation studies, after RNA FISH, rabbit anti-Nucleolin was used to detect co-localisation of NLC1-C with Nucleolin in NT2 cells. Images were taken with an Olympus IX70 DeltaVision RT Deconvolution System microscope. All experiments were repeated at least three times. The information on antibodies is provided in Supplementary Table S4.

### Plasmid construction

For pcDNA3.1-NLC1-C (NLC1-C), NLC1-C was inserted into the *BamHI*/*NotI* sites of the pcDNA3.1 vector, which was kindly provided by Mian Wu (University of Science and Technology of China (USTC), Hefei, Anhui, China). The primers for pcDNA3.1-NLC1-C construction are listed in Supplementary Table S1. The NLC1-C shRNA oligos were inserted into the *AgeI* and *EcoRI* sites of PLKO.1 vector. The sequences of the NLC1-C shRNA oligos are presented in Supplementary Table S3. The various Nucleolin constructs, inserted into pEGFP-C1, were kindly provided by Anjana Saxena (Brooklyn College, New York, NY, USA). The Nucleolin-FLAG expression vector was constructed by cloning human Nucleolin cDNA into the p3XFLAG-myc-CMV™-24 expression vector at the *EcoRI* and *SalI* sites. For the probe vector for ISH, a 360 bp fragment of NLC1-C was inserted into the *HindIII*/*EcoRI* sites of the pSPT18/19 plasmid. The NLC1-C vector used for RNA pull-down experiments was constructed by cloning the whole NLC1-C sequence into the pSPT18/19 plasmid at the *HindIII*/*EcoRI* sites. The NLC1-C vector used for NB was constructed by cloning the whole NLC1-C sequence into the pEASY- T3 Vector using pEASY-T3 Cloning Kit. WT NLC1-C-pMIR-Report was obtained by amplifying a 730-bp fragment of NLC1-C harbouring the miR-320a and miR-383-binding sites, whereas mutated NLC1-C-pMIR-Report was generated by PCR-based site-directed mutagenesis. WT and mutated NLC1-C-pMIR-Report sequences were inserted into the *HindIII* and *SpeI* sites of the pMIR-Report vector. WT miR320 promoter and miR-383 promoter were obtained by amplifying two −1000 /+50 bp fragments of miR320 promoter and miR-383 promoter harbouring the NLC1-C-binding sites, whereas mutated miR320 promoter and miR-383 promoter were generated by PCR-based site-directed mutagenesis. WT and mutated miR320 promoter and miR-383 promoter sequences were inserted into the *KpnI* and *XhoI* sites of the pGL3-basic vector. PCR was performed with Phanta Super-Fidelity DNA Polymerase (Vazyme Biotech, Piscataway, NJ, USA). All primers for plasmid construction are listed in Supplementary Table S1.

### Statistical analysis

Statistical analysis was carried out using Microsoft Excel software and GraphPad Prism to assess differences between experimental groups. Statistical significance was analysed by Student's *t*-test and expressed as a *P*-value. P-values lower than 0.05 were considered to be statistically significant. One asterisk, two asterisks, and three asterisks indicate *P*<0.05, *P*<0.01, and *P*<0.001, respectively.

## Figures and Tables

**Table 1:** Location of NLC1-C in the testes of infertile men with maturation arrest (MA)

*Testicular tissue (ages)*											
RNA	M350 (30)	M351 (27)	M352 (21)	M353 (35)	M354 (21)	M357 (25)	M361 (26)	M362 (27)	M363 (33)	M365 (29)	M398 (27)
NLC1-C	C	N	N	N	N	N	N	C	N	N	N

Location of NLC1-C in the testes of infertile men with MA was examined by RNA fluorescence ISH on cryosections analysis. M350(30) means that the testes of infertile men with MA whose age was 30 was numbered 350. C means that the expression of NLC1-C was primarily located in the cytoplasm of spermatogonia and primary spermatocytes. N means that the expression of NLC1-C was primarily located in the nucleus of spermatogonia and primary spermatocytes.

## Abbreviations

NLC1-C: narcolepsy candidate-region 1 genes
miRNA: microRNA
lncRNAs: long non-coding RNAs
MA: maturation arrest
Hypo: hypospermatogenesis
qRT-PCR: quantitative real-time PCR
LINC00162: long intergenic non-protein-coding RNA162
NOA: nonobstructive azoospermia
siRNAs: small interfering RNAs
RISC: RNA-induced silencing complex
NT2: NTERA-2
NB: northern blot
DIG: digoxigenin
ChIP: Chromatin IP
CCK-8: Cell Counting Kit-8
ISH: *in situ* hybridisation
