# Supplementary material for: Downregulation of miR-320a/383-sponge-like long non-coding RNA NLC1-C (narcolepsy candidate-region 1 genes) is associated with male infertility and promotes testicular embryonal carcinoma cell proliferation
Source: Cell Death Dis. 2015 Nov 5;6(11):e1960–. doi: 10.1038/cddis.2015.267 (PMC4670917; doi:10.1038/cddis.2015.267)
Supplement: Supplementary Information [file cddis2015267x1.pdf]

Supplementary information:

Downregulation of miR-320a/383-sponge-like long non-coding RNA NLC1-C (narcolepsy candidate-region 1 genes) is associated with male infertility and promotes testicular embryonal carcinoma cell proliferation

Mingrong Lü [a,b], Hui Tian [a,e], Yun-xia Cao[b], Xiaojin He[b], Lingling Chen[c], Xiaoyuan Song [e], Ping Ping [d], Hefeng Huang [a,c] *and Fei Sun [a,c] *

Supplementary Figure S1

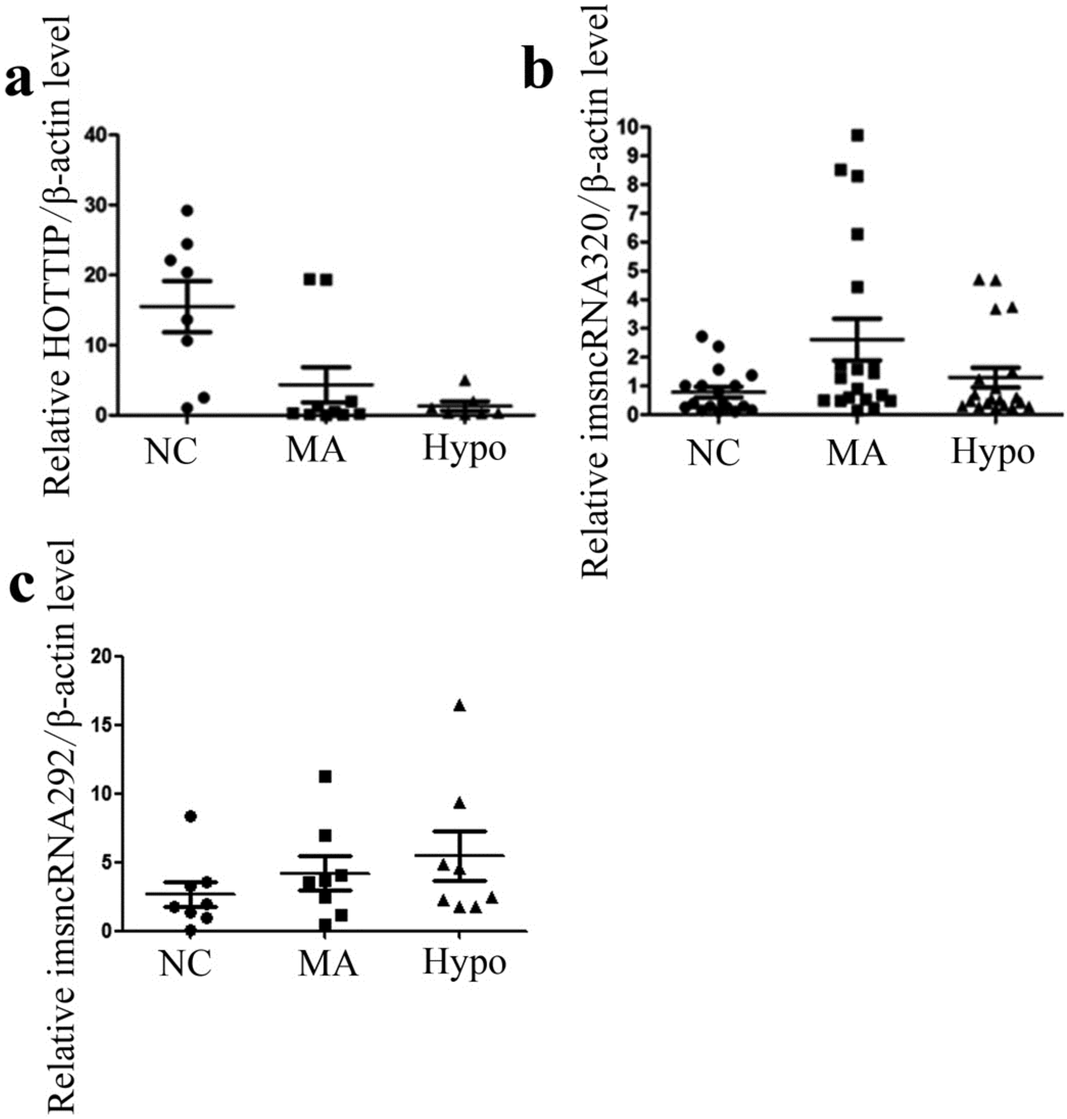

Figure S1. Confirmation of microarray data by quantitative real-time PCR. Quantitative real-time PCR analysis confirmed microarray data: lncRNA HOTTIP was down-regulated and imsncRNA320 and imsncRNA292 was up-regulated in NOA (including MA and Hypo) patients. NOA : non-obstructive azoospermia; MA: maturation arrest; Hypo: Hypospermatogenesis. Error bars indicate the SEM.

Supplementary Figure S2

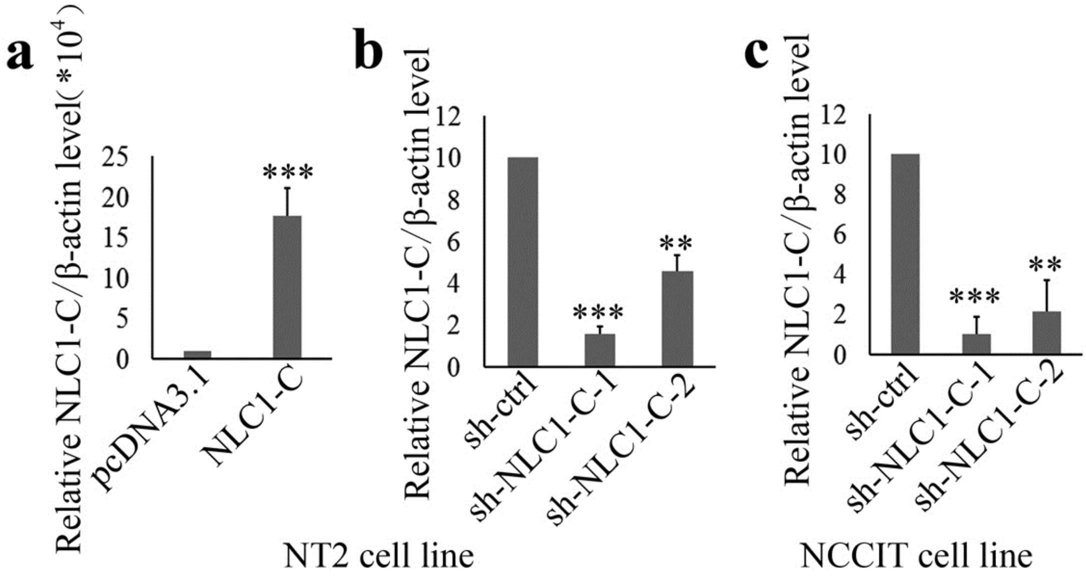

Figure S2. Real-time PCR analysis of NLC1-C expression in NT2 and NCCIT cells. a. Real-time PCR analysis of NLC1-C expression in NT2 cells when transfected with pcDNA3.1 or pcDNA3.1-NLC1-C (NLC1-C) for 48h. b and c. Real-time PCR analysis of NLC1-C expression in NT2 (b) and NCCIT (c) cells when infected with control shRNA or NLC1-C-1/2 shRNA lentiviral particles culture for 48h.

Supplementary Figure S3

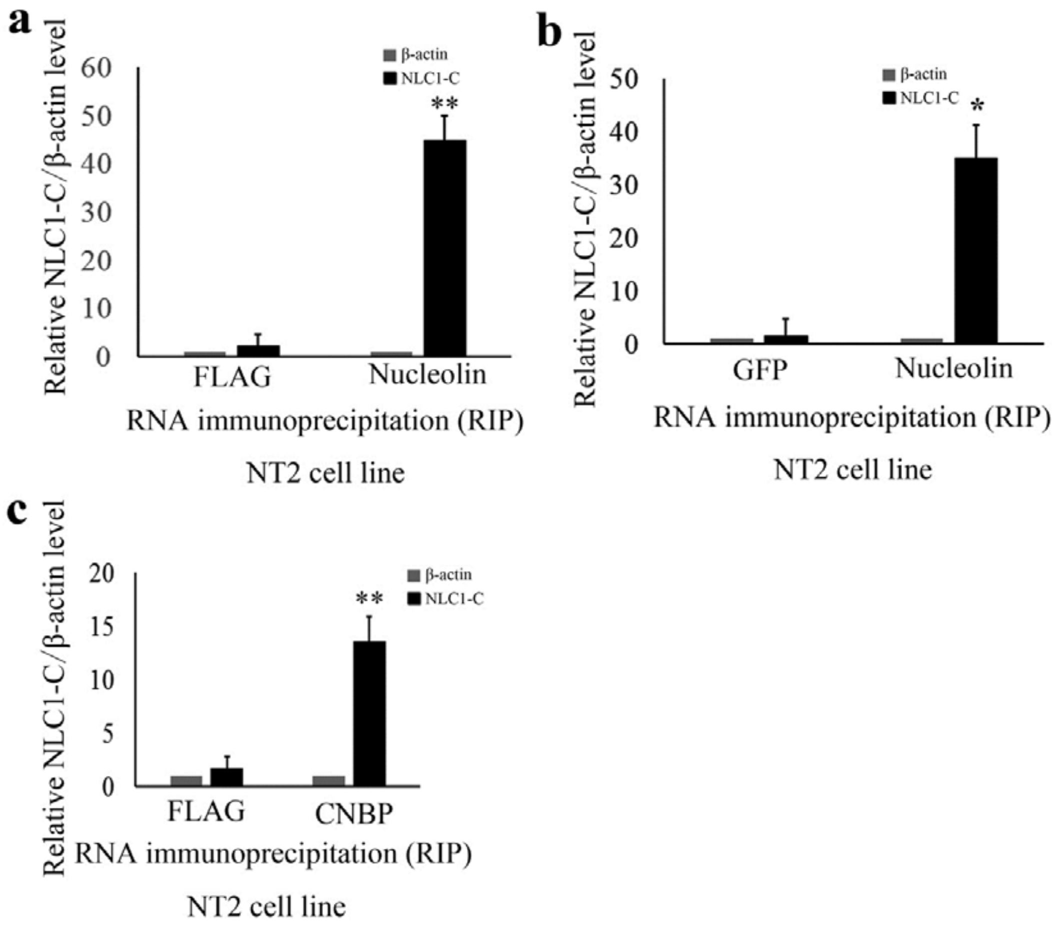

Figure S3. Nucleolin IP and CNBP IP specifically retrieves NLC1-C RNA in vitro.

a, b and c. The indicated plasmids were transfected into NT2 cells for 48 h. Lysates were prepared from these cells. RNAs that were co-precipitated with FLAG, GFP were detected by qRT–PCR using primers for NLC1-C and β-actin (as negative control). IP enrichment was determined as the amount of RNA associated to the various proteins. Data are from one of three independent experiments and are represented as mean±s.d. with n=3. Data (mean±s.d, n = 3 replicates) is relative to mock-IP (FLAG or GFP)

Supplementary Figure S4

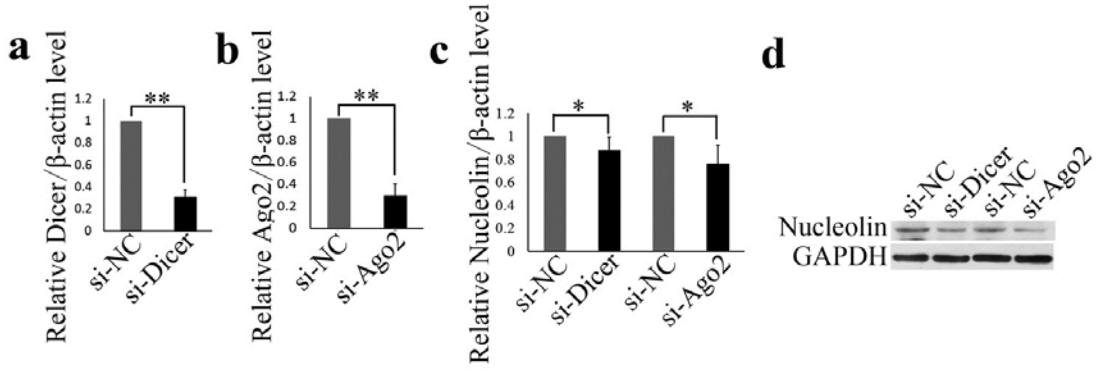

Figure S4. Knockdown of Dicer and Ago2 decreased Nucleolin expression. a, b ,c and d. NT2 cells were transfected with control siRNA (si-NC) or Dicer or Ago2 siRNAs separately. The expression of Dicer(a), Ago2(b), Nucleolin(c and d) ware detected by quantitative real-time PCR and western blot after transfection for 48 h. GAPDH was used as internal reference. Data are represented as mean±s.d. from three independent experiments. *P<0.05 or **P<0.01 compared with control RNA.

Supplementary Figure S5

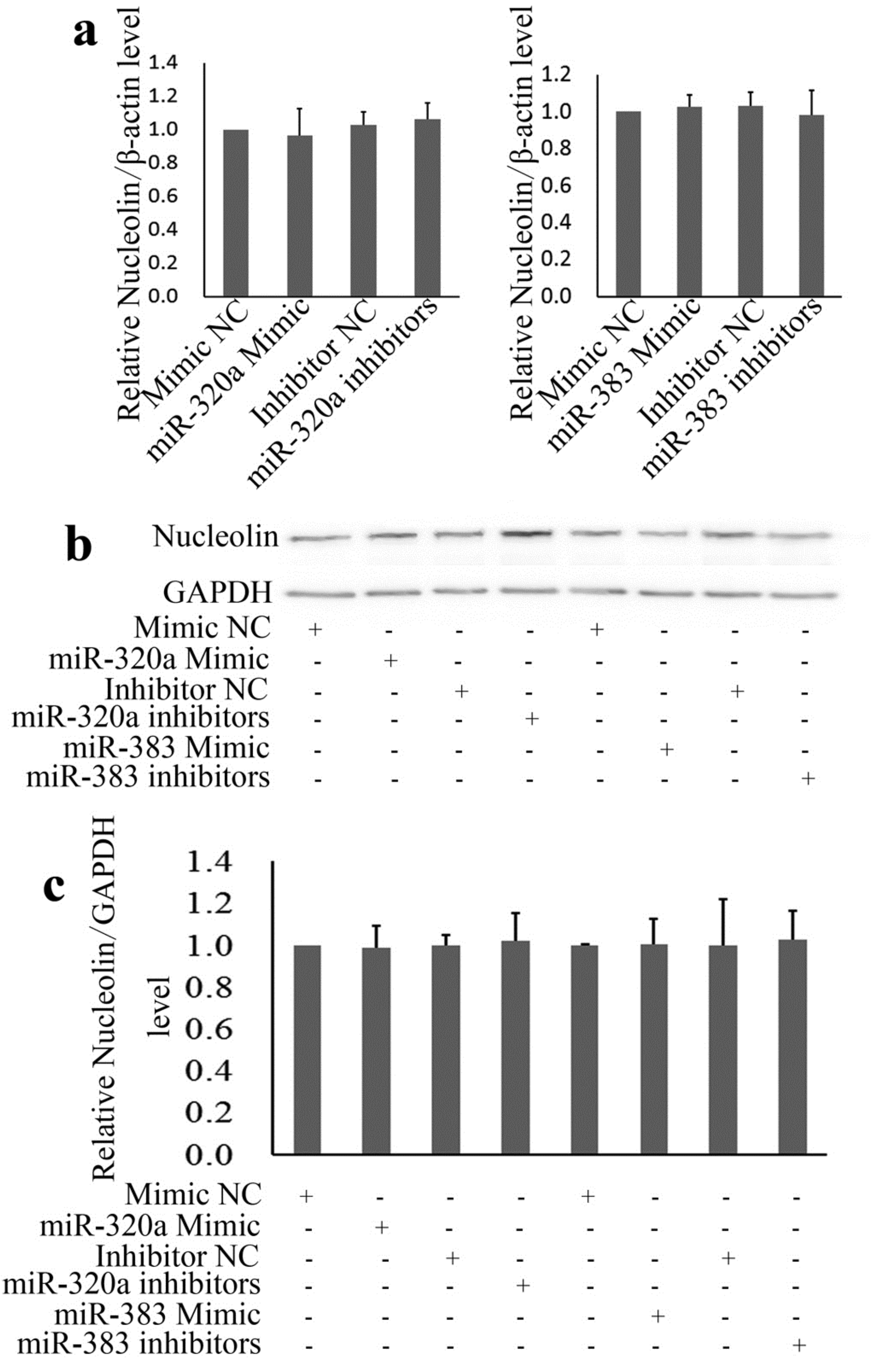

Figure S5. Nucleolin was not the target of miR-320a and miR-383. a and b. Quantitative

real-time PCR(a) and western blot(b)analysis of Nucleolin expression after NT2 cells were transfected with indicated miRNAs for 48 h and the corresponding densitometric analysis (c). Data are represented as mean±s.d. from three independent experiments.

Supplementary Table S1. List of primer pairs for construction of vectors.

Name of gene	Sequence (5' to 3')	Annealing temperature (°C)
**NLC1-C-pcDNA3.1**	Forward:ATTAGGATCCTGAAGTGCTGACGGGTGGAGCGA<br>Reward:CGCTGCGGCCGCTTTCCCACTGACTTGTTATATTT	58
**NLC1-C (ISH, sense）**	Forward:ACTGAAGCTTCTCTATGACCTCATCCATGGGAAA<br>Reward:AGCTGAATTCTCCCACTGACTTGTTATATTTTCA	65
**NLC1-C (ISH, antisense**	Forward:ACTGAAGCTTTGGAGCCTCATTCACTGTGAAGT<br>Reward:AGCTGAATTCCTCTATGACCTCATCCATGGGAAA	64
**NLC1-C (Pulldown)**	Forward:ACTAAAGCTTTGAAGTGCTGACGGGTGGAGCGA<br>Reward:ACGAATTCTTTCCCACTGACTTGTTATATTTTCA	60
**NLC1-C (miR-320a binding site mutation)**	Forward:ATGGAAGAGTGCTAACTATAGGTGGGGAGCACATGTGGTGAAGAT<br>Reward: TAGTTAGCACTCTTCCATCTGTGGGGGTCGTCCCTACT	65
**NLC1-C (miR-383 binding site mutation)**	Forward:TTGACATACGAGAGACTAGATCAGTCACTTCACAGTGAATGAGGCT<br>Reward:ATCTAGTCTCTCGTATGTCAACCTGCTCCTTCAGGTCCCTCT	64
**NLC1-C-pZW1-snovector**	Forward: ATTGGAATTCTGAAGTGCTGACGGGTGGAG<br>Reward: ACGGGGTACCTTTCCCACTGACTTGTTATAT	62
**Nucleolin-FLAG**	Forward:AGCTGAATTCATGGTGAAGCTCGCGAAGGCAGGTAAAAA<br>Reward: TGCAGTCGACCTATTCAAACTTCGTCTTCTTTCCTTGT	58

Supplementary Table S2. List of primer pairs for real-time PCR and PCR.

Name of gene	Forward primer sequence (5' to 3')	Reverse primer sequence (5' to 3')
**β-actin(human)**	TGGCACCCAGCACAATGAA	CTAAGTCATAGTCCGCCTAGAA
**NLC1-C**	TGCCTGGACTTTCAAGAGGTAA	GCTCTCAGTCAGCAGACACTT
**Nucleolin**	CGTTCGGGCAAGGATAGTTAC	GGCATCCTCCTCACTGTTGAAG
**Ims320**	ATTTGAGGTATAAAAATTATAACTAT	TTATAAGTCATTCGTGCCACTTCTG
**HOTTIP**	CCTAAAGCCACGCTTCTTTG	TGCAGGCTGGAGATCCTACT
**pri-miR-383**	CTTTCCCAAGAGTTTCACT	CCACTCCAGTCCACCAAAT
**pri-miR-320a**	TGGGCCACAGTATTTATCAGGCGGCGCTT	AGCGCCGCAGCATAGCGTAGCCCAGACCA
**miR-320 promotor**	CACAGAGCCTCGAATGCCATACCTAGGATTCATC	ACCTCATCCTTTTTCGCCCTCTCAACCCAGCTTT
**SGCZ promotor**	AAACTATAAAGAACTAGGGGGAGAGACAGCTGTC	CAGTGGGGAAAAGAAGATAATCAAGTCAGAAGGG

Supplementary Table S3. Sequence of oligonucleotides.

Oligonucleotides	Sequence (5' to 3')
**Inhibitor NC**	CAGUACUUUUGUGUAGUACAA
**has-miR-320a inhibitors**	UCGCCCUCUCAACCCAGCUUUU
**has-miR-383 inhibitors**	AGCCACAAUCACCUUCUGAUCU
**Mimics NC**	UUCUCCGAACGUGUCACGUTT<br>ACGUGACACGUUCGGAGAATT
**hsa-miR-320a mimics**	AAAAGCUGGGUUGAGAGGGCGA<br>GCCCUCUCAACCCAGCUUUUUU
**hsa-miR-383 mimics**	AGAUCAGAAGGUGAUUGUGGCU<br>CCACAAUCACCUUCUGAUCUUU
**LNA miR-320**	TCGCCCTCTCAACCCAGCTTTT
**LNA miR-383**	AGCCACAATCACCTTCTGATCT
**NLC1-C-PLKO.1-1**	GCTGGCTCACCCTGGCACTGCA
**NLC1-C-PLKO.1-2**	GACCGTCCCTGGCCGCCAAGTG
**NLC1-C (RNA-FISH)-sense**	UCAAGAGGUAAAUUCCGAGCUCUGCUCUGAGGCCU
**NLC1-C (RNA-FISH)-antisense**	AGGCCUCAGAGCAGAGCUCGGAAUUUACCUCUUGA

Supplementary Table S4. List of primary antibodies and secondary antibodies.

Antigen	WB dilutions	Antibody name	Catalog number	Company
GAPDH	1:1000	GAPDH (14C10) Rabbit mAb	#2118	cell signaling technology
Nucleolin	1:1000	Rb-pAb to Nucleolin	ab70493	abcam
c-Myc	1:200	c-Myc Rabbit Ab	#9402	cell signaling technology
Rabbit IgG secondary antibody	1:6000	Rabbit IgG secondary antibody - H&L	ab6802	abcam
Mouse IgG secondary antibody	1:6000	Mouse IgG secondary antibody - H&L	ab6819	Abcam
PARP	1:200	PARP (46D11) Rabbit mAb	#9532S	cell signaling technology
Caspase-3	1:6000	Caspase-3 (8G10) Rabbit mAb	#9665S	cell signaling technology
Caspase-8	1:6000	Caspase-8 (D35G2) Rabbit mAb	#4790S	cell signaling technology
Caspase-9	1:6000	Cleaved Caspase-9 (Asp330) (D2D4) Rabbit mAb	#7237S	cell signaling technology
Anti-Digaoxingenin -Fluorescein, Fab fragments	1:250	Anti-Digaoxingenin -Fluorescein, Fab fragments	11207741910	Roche
